# The hypointense pulvinar sign on susceptibility weighed magnetic resonance imaging: A visual biomarker for iron deposition in epilepsy

**DOI:** 10.1177/19714009241303050

**Published:** 2024-12-02

**Authors:** Victoria Martella, Riccardo Ludovichetti, Nathalie Nierobisch, Carina Obermüller, Felix Gunzer, Fabienne Maibach, Philip Heesen, Qeumars Hamie, Robert Terziev, Marian Galovic, Zsolt Kulcsar, Nicolin Hainc

**Affiliations:** 1 Department of Neuroradiology, Clinical Neuroscience Center, 27243 University Hospital Zurich, University of Zurich, Switzerland; 2 Department of Neurology, Clinical Neuroscience Center, 27243 University Hospital Zurich, University of Zurich, Switzerland

**Keywords:** Epilepsy, pulvinar, thalamus, magnetic resonance imaging, iron

## Abstract

**Objective:**

Our study aimed to investigate potential alterations in iron deposition within pulvinar, using susceptibility weighted imaging (SWI) MRI in epilepsy patients through a biomarker termed the “hypointense pulvinar sign.”

**Methods:**

A full-text radiological information system search of radiological reports was performed for the term “epilepsy” between 2014 and 2022. Only patients with the diagnosis of epilepsy were included. SWI was assessed by two readers recording lateralization of an asymmetrically more hypointense pulvinar. Cohen’s kappa for inter-rater reliability was calculated. Fisher’s exact test was performed to assess for significance between groups.

**Results:**

Our epilepsy cohort comprised 105 patients with following diagnoses: 45 intra-axial tumor, 13 meningioma, 13 MRI negative, 12 encephalomalacia, seven siderosis, six cavernoma, five arteriovenous malformation, two acute demyelinating encephalomyelitis, one tuberous sclerosis, one giant aneurysm. The hypointense pulvinar sign was correct in 44% of cases. Notably, right hemispheric lesions exhibited a significantly higher proportion of correct hypointense pulvinar signs compared to the left hemisphere (46% vs 24%; *p* = 0.044). Inter-rater reliability was substantial at 0.62 (*p* < 0.001). Only two of 21 (10%) of healthy controls demonstrated a hypointense pulvinar sign, which was significantly different from the epilepsy cohort (*p* < 0.01).

**Conclusions:**

The hypointense pulvinar sign has proven to be a reproducible, simple to use biomarker for iron deposition in epilepsy which could be considered for inclusion into multimodal precision medicine models.

## Introduction

A seizure is characterized by abnormal and uncontrolled neuronal activity, presenting with a spectrum of symptoms from focal seizures to generalized status epilepticus. On diffusion weighted imaging (DWI) MRI, cortical restricted diffusion is the most common seizure-related finding,^1^ observed either within specific affected brain regions or diffusely over an entire hemisphere. Neuronal swelling, which is the endpoint of various pathomechanisms seen in the setting of seizures,^2–5^ brings about the restricted diffusion seen on MRI. Notably, restricted diffusion is also found in the hippocampus and the pulvinar of the thalamus, as these structures are frequently involved during a seizure.^2,6,7^ The pulvinar plays a central role in seizure initiation and propagation^8–10^ attributed to its extensive connections with the ipsilateral cingulate, parietal, temporal (mesial and lateral), prefrontal, and occipital cortices.^11–14^

Beyond neuronal swelling, seizure activity has been associated with iron deposition,^15,16^ yet little is known regarding iron metabolism and its role in the epileptogenic brain. Studies on post status epilepticus rats have demonstrated higher hippocampal iron levels^17^ and pathological iron deposits in thalamic nuclei.^18^ In a study involving human brain specimens, iron retention was demonstrated in the hippocampus of patients with status epilepticus and temporal lobe epilepsy.^17^ Moreover, using the advanced MRI surrogate marker for iron, quantitative susceptibility mapping (QSM), increased iron accumulation was identified in the seizure-onset zone in epilepsy patients through comparison of inter-ictal and postictal states.^17^ A further MRI-based study revealed, in addition to the expected increase in cortical iron, a concurrent decrease in subcortical iron (putamen, globus pallidus, substantia nigra, and red nucleus),^19^ suggesting an epilepsy-induced re-distribution of brain iron. Notably, this study did not assess the thalamus. In clinical practice, susceptibility weighted imaging (SWI) MRI has demonstrated very high sensitivities to iron^20^ and serves as a tool to assess iron in the form of hemosiderin, ferritin, and deoxyhemoglobin.^21,22^

Epilepsy appears to be a progressive condition.^23–25^ Accelerated whole-brain cortical thinning was found in patients with focal epilepsy compared to age- and sex-matched healthy volunteers, independent of seizure frequency, duration, or antiepileptic drug load.^26^ Similar whole-brain findings have been reported in seizure-free temporal lobe epilepsy patients.^27^ These observations underscore underlying pathological mechanisms driving progressive, whole-brain changes in epilepsy patients independent of seizure frequency, which has led to a recent paradigm shift in the understanding of epilepsy towards a network disease as opposed to a symptom of local brain abnormalities.^28,29^ Further corroborating this notion is a study demonstrating that successful epilepsy surgery (i.e., seizure-freedom) halted the accelerated rate of cortical thinning, suggesting an interruption of the epilepsy network.^30^ The goal of this study was to explore potential alterations in iron deposition within pulvinar of the thalamus, using susceptibility weighted imaging (SWI) MRI in patients with epilepsy, irrespective of seizure type or frequency.

## Materials and methods

### Patient cohort

**Figure 1.**
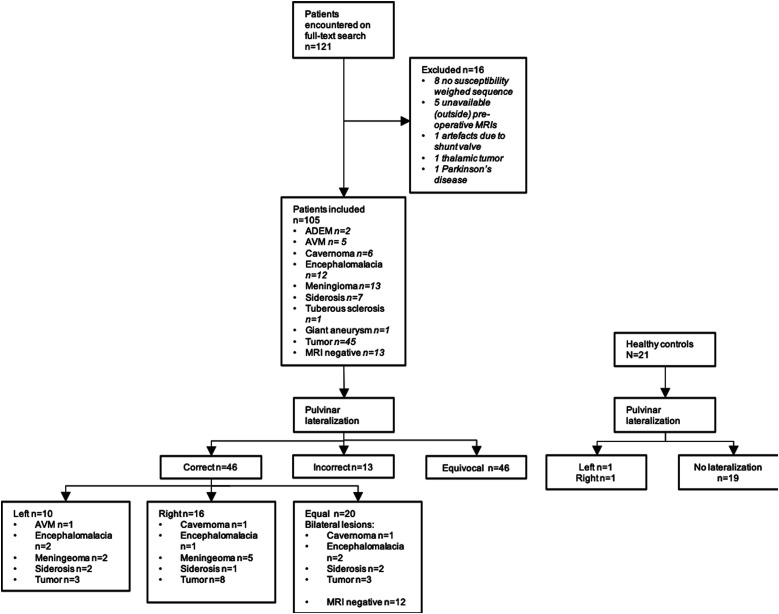
Flow-diagram of patients included in the study.

Ethical approval was obtained through the Institutional Review Board (Kantonale Ethikkommission Zuerich, BASEC Nr. 2022-00041) prior to commencing the study. Informed consent was obtained for all patients. A full-text radiological information system search of radiological reports was performed for the term “epilepsy” between 2014 and 2022. Only patients with the diagnosis of epilepsy were included (Figure 1). Patients where the outside MRI performed at time of epilepsy diagnosis was not available in our PACS were excluded. Also excluded were patients whose MRI protocols did not include susceptibility weighted sequences, MRIs that were quality-degraded due to artifacts, and patients in which the primary brain lesion involved the thalamus. Finally, patients with Parkinson’s disease were excluded as differences in the pulvinar susceptibility have been demonstrated in a previous study.^31^

Diagnosis of epilepsy was established by experienced neurologists specializing in epilepsy. Workup included clinical, electroencephalography (EEG), and neuroimaging data.

For healthy controls, patients without documented history of epilepsy, abnormal brain findings, or systemic disease were included. 

### MRI findings

**Figure 2.**
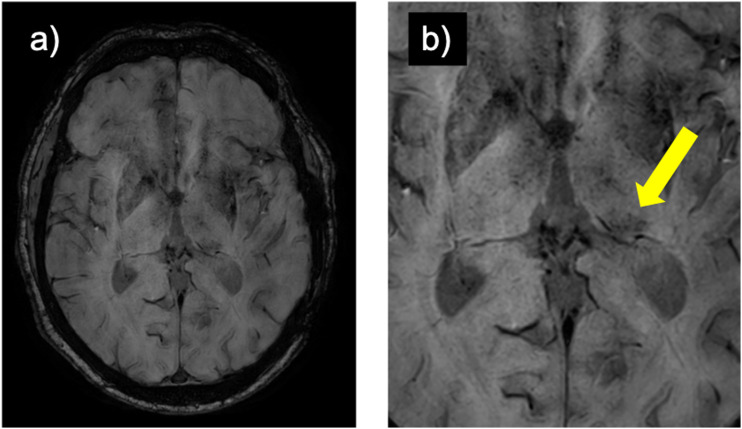
(a) whole-brain susceptibility weighted image and (b) zoomed-in image of a left-sided hypointense pulvinar sign (arrow) in a patient with post-traumatic left temporal encephalomalacia (not shown).

MRI of the brain was routinely performed with administration of intravenous contrast and the entire protocol consisted of axial FLAIR, T2, DWI, SWI, and 3D T1 MPRAGE pre- and post-gadolinium, as per institutional protocol. Initial MRI performed at the time of patient presentation and the most recent (i.e., longest term) follow-up MRI were assessed. Axial susceptibility-weighed sequences were independently assessed by two readers (VM and NH), with one and 8 years of neuroradiology reading experience. Disagreements were settled by a third reader with 3 years of neuroradiology reading experience (NN) Lateralization of hypointensity (i.e., asymmetrically more hypointense) of the pulvinar nucleus was recorded (left, right, and equal) in a blinded manner (Figure 2).

The pulvinar rating was deemed to be “correct,” “equivocal,” or “incorrect” based on correlation with the underlying brain lesion (e.g., tumor) identified on MRI:

(1) Correct: “Correct” lateralization denoted an asymmetrically more hypointense pulvinar on SWI found ipsilateral to the underlying brain lesion. Furthermore, a pulvinar rating of “equal” in the context of MRI-negative epilepsy or lesions involving both cerebral hemispheres was also considered “correct.”
(2) Equivocal: A pulvinar rating of “equal” in the context of a single, unilateral brain lesion seen was considered “equivocal.”
(3) Incorrect: A contralateral hypointense pulvinar with respect to a single brain lesion or an asymmetric pulvinar in MRI-negative epilepsy/bilateral hemispheric brain lesions was considered “incorrect.”

### Statistical analysis

Numerical variables are presented as median (1^st^ quartile, 3^rd^ quartile) while categorical variables are presented as *n* (%). Cohen’s kappa for inter-rater reliability was calculated.^32,33^ Fisher’s exact test was performed to test for differences between groups. All statistical analyses were conducted using R statistical software. A *p*-value <0.05 was considered statistically significant.

## Results

### Patient cohort

121 patients were encountered. 16 were excluded due to following reasons: eight did not have susceptibility weighted sequences in their MRI protocol; 5 with outside pre-operative MRIs not found in our PACS; one due to artefacts from the ventriculoperitoneal shunt valve limiting assessment of the pulvinar; one tumor involving the thalamus; one patient with Parkinson’s disease. The final epilepsy cohort comprised 105 patients (38 female, 67 male; median age 48 years, range 18 to 85 years). This was supplemented with 21 healthy controls (15 female, six male; median age 49 years, range 25 to 66).

Seizure semiology and EEG data was available for review in all cases. Focal epilepsy was diagnosed in 73 patients (31 with secondary generalization). Primary generalized seizures were diagnosed in 31 patients. One patient had a documented episode of status epilepticus without further description of seizure semiology, and a total of six patients included had a documented episode of status epilepticus. Lateralization on inter-ictal EEG was found in 23 patients. There were no statistical differences between the correct and equivocal/incorrect groups in terms of seizure semiology or the presence of lateralization on EEG. 

## MRI Findings

### Initial MRI

MRI performed at diagnosis of epilepsy was predominantly on 3T scanners 100/105 (95%); the remainder were scanned on 1.5 T (5%). The Susceptibility Weighed Imaging (SWI) sequence (Siemens Healthineers, Erlangen, Germany, and Philips Healthcare, Amsterdam, Netherlands) was performed in 93/105 (88%) cases, the remaining 12 (12%) were Susceptibility Weighted Angiography (SWAN) on GE (GE Healthcare, Chicago, USA).

Diagnoses included intra-axial tumor *n* = 45 (43%; 14 oligodendroglioma, 13 astrocytoma, 7 glioblastoma, 7 metastatic disease, 3 ganglioglioma, and 1 dysembryoplastic neuroepithelial tumor), meningioma *n* = 13 (12%), MRI negative *n* = 13 (12%; one autoimmune encephalitis, 12 cryptogenic of which inter-ictal EEG revealed no lateralizing focus in 9/12 cases), encephalomalacia *n* = 12 (11%; seven post-ischemic, five post-traumatic), siderosis *n* = 7 (7%, all due to ruptured aneurysm), cavernoma *n* = 6 (6%), arteriovenous malformation (AVM) *n* = 5 (5%), acute demyelinating encephalomyelitis (ADEM) *n* = 2 (2%), tuberous sclerosis *n* = 1 (1%), giant aneurysm compressing the temporal lobe *n* = 1 (1%).

MRI performed upon patient presentation demonstrated a correct hypointense pulvinar sign in 46 (44%) of cases (Table 1). The highest proportion of correct cases was seen in MRI-negative patients (92%), followed by siderosis (71%), meningioma (54%), encephalomalacia (42%), cavernoma (33%), tumor (31%), and AVM (20%) (Table 2). ADEM, tuberous sclerosis, and giant aneurysm did not demonstrate any cases of correct pulvinar lateralization and were only present in small numbers. Incorrect pulvinar lateralization was found in 13 (12%) cases, while the sign remained equivocal in 46 (44%) cases.

**Table 1.** MRI findings grouped by pathological lesion on initial and follow-up MRIs.

Lesion	Initial MRI	Lateralization of lesion on MRI			Lateralization of hypointense pulvinar			Follow-up MRI	Lateralization change on follow-up MRI	
	*n* = 105	Left *n* = 46	Right *n* = 36	Bilateral/None *n* = 23	Correct *n* = 46	Incorrect *n* = 13	Equivocal *n* = 46	*n* = 84	Incorrect/equivocal to correct *n* = 10	Correct to incorrect/equivocal *n* = 10
ADEM	2	2	0	0	0	0	2	2	0	0
AVM	5	4	1	0	1	1	3	3	1	0
Cavernoma	6	2	3	1	2	0	4	6	1	2
Encephalomalacia	12	7	3	2	5	3	4	6	1	1
Meningioma	13	6	7	0	7	1	5	12	0	5
Siderosis	7	4	1	2	5	0	2	1	1	0
Tuberous sclerosis	1	0	0	1	0	1	0	1	0	0
Giant aneurysm	1	0	1	0	0	0	1	1	1	0
Tumor	45	21	20	4	14	6	25	44	5	1
MRI negative	13	0	0	13	12	1	0	8	0	1

**Table 2.** Sub-analysis of pulvinar lateralization based on pathological entity.

Lesion	Pulvinar correctly lateralized			Total (%)
	Left	Right	Equivocal	
AVM	1			
Percent of total cases	20%			20
Cavernoma		1	1	
Percent of total cases		16.6%	16.6%	33.3
Encephalomalacia	2	1	2	
Percent of total cases	16.6%	8.3%	16.6%	41.6
Meningioma	2	5		
Percent of total cases	15.3%	38.4%		53.8
Siderosis	2	1	2	
Percent of total cases	28.5%	14.2%	28.5%	71.4
Tumor	3	8	3	
Percent of total cases	6.6%	17.7%	6.6%	31.1
Unremarkable			12	
Percent of total cases			92.3%	92.3

**Figure 3.**
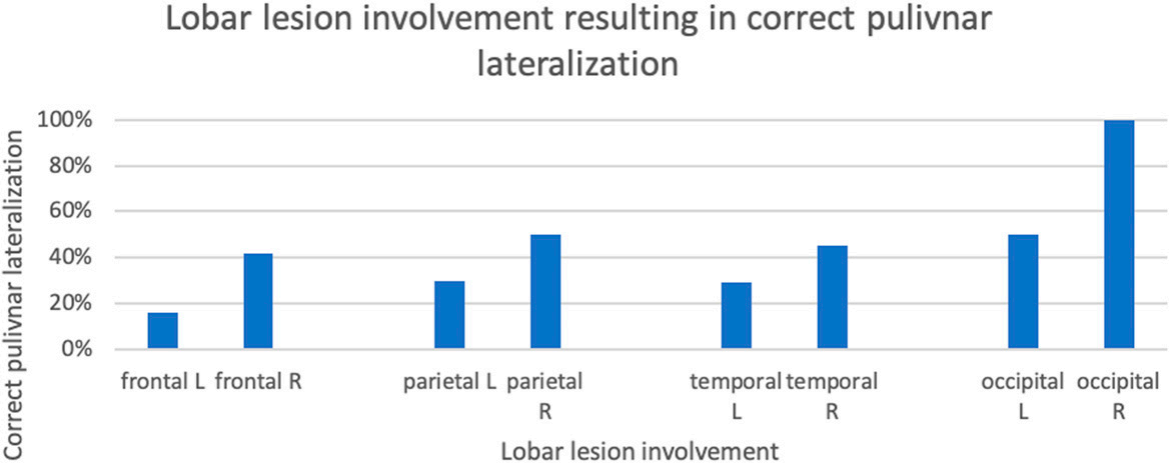
Bar chart displaying lobar lesion involvement resulting in correct lateralization (left (L), right (R)) of the hypointense pulvinar sign, expressed as a percentage of total number of lobes involved per hemisphere.

Upon sub-analysis of patients with lesional MRI, a correct hypointense pulvinar sign based on lobar involvement of the underlying lesion was determined as follows: left frontal (4/25), right frontal (8/19), left parietal (3/10), right parietal (4/8), left temporal (5/17), right temporal (5/11), left occipital (1/2), right occipital (1/1) (Figure 3). This totaled to 13/54 (24%) on the left and 18/39 (46%) on the right which proved to be statistically significant (*p* = 0.044).

Healthy controls were scanned predominantly on 3T scanners 19/21 (90%); the remainder were scanned on 1.5 T (10%). The SWI sequence was performed in 15/21 (71%) cases, the remaining 6 (29%) were SWAN. Only 2/21 (10%) of healthy controls demonstrated a lateralized hypointense pulvinar, the remainder (19/21; 90%) were not lateralized. The difference between correct pulvinar signs in epilepsy patients and a lateralized hypointense pulvinar in healthy controls was statistically significant (*p* = 0.0028).

### Follow-up MRI

84 of 105 patients had a follow-up MRI at a median interval of 117 weeks (1^st^ quartile, 3^rd^ quartile; 68, 256). These included patients with the following initial pulvinar lateralization results: 34 correct, 9 incorrect, and 41 equivocal.

**Figure 4.**
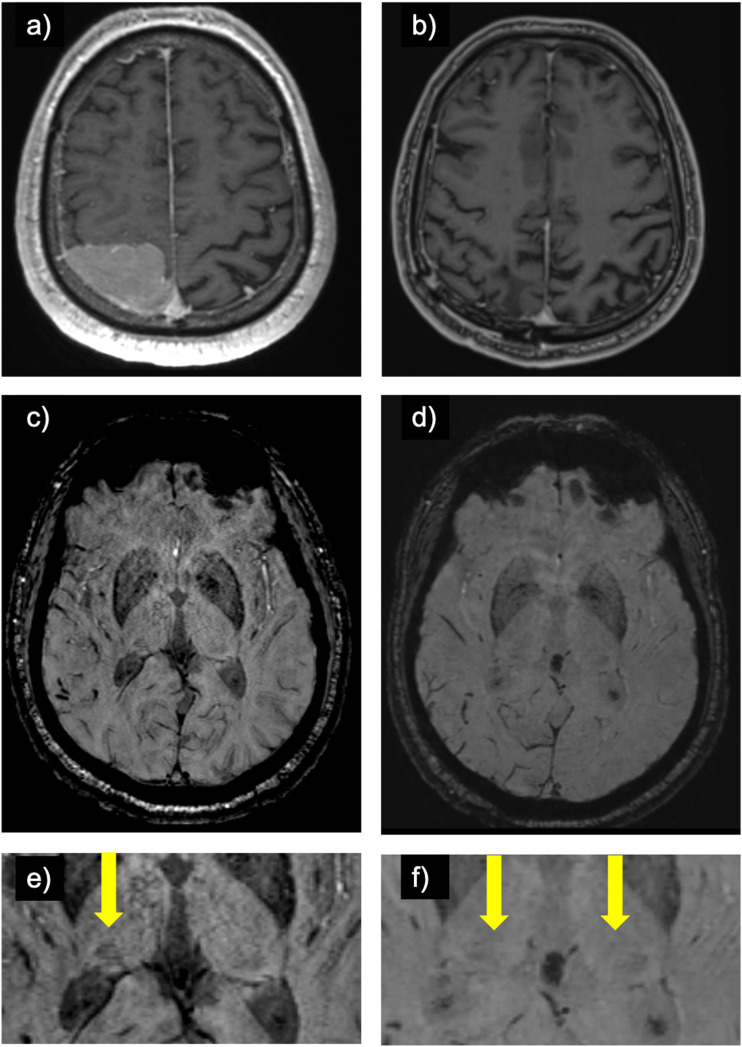
Post glioblastoma resection development of the hypointense pulvinar sign: (a) contrast-enhanced T1-weighted image of a patient with a left temporal glioblastoma. (b) symmetrical hypointensity of the pulvinar on susceptibility weighted imaging on early post-resection MRI (“equal”; arrows on zoomed-in image c)) which evolved to a left-sided hypointense pulvinar sign upon further follow-up (d; arrows on zoomed-in image e)).

**Figure 5.**
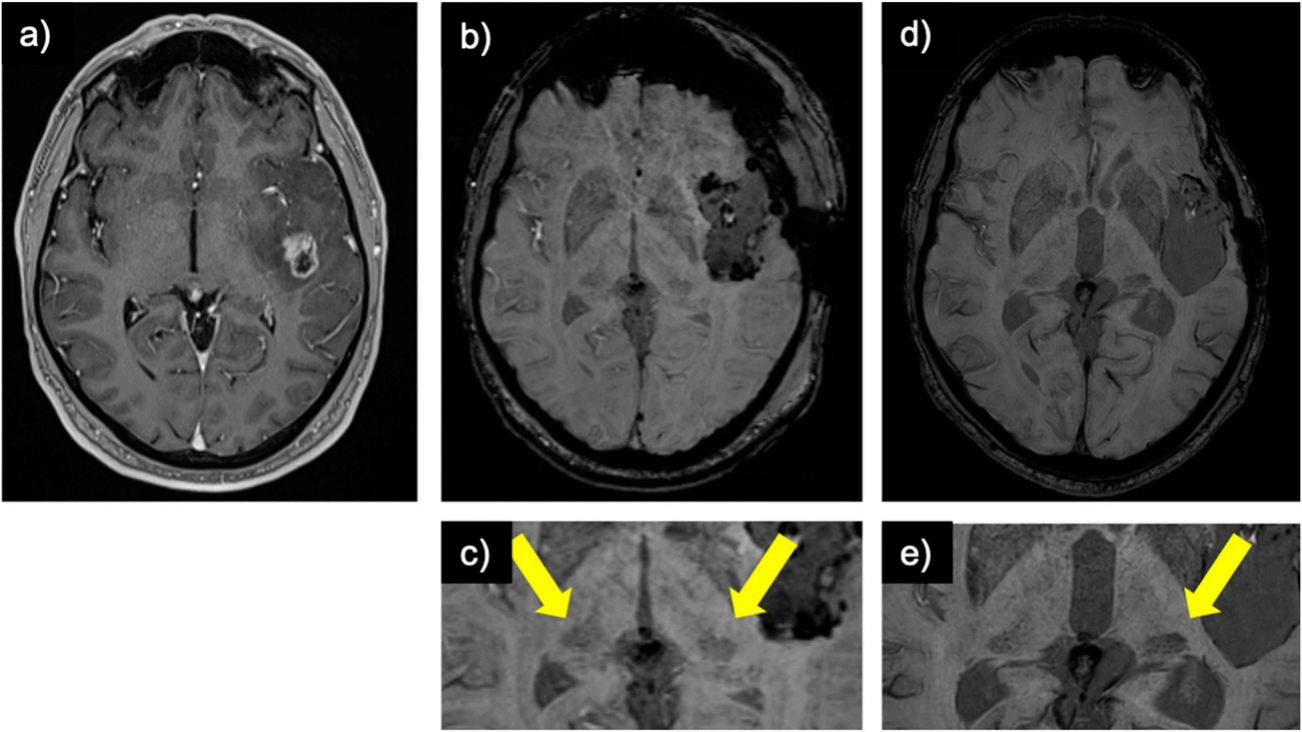
Post meningioma resection resolution of the hypointense pulvinar sign: (a) contrast-enhanced T1-weighted image of patient with a meningioma over the right parietal lobe and (b) status post-resection. (c) whole-brain susceptibility weighted image pre-resection and (d) post-resection. (e) zoomed-in susceptibility weighted image pre-resection demonstrating a right-sided hypointense pulvinar sign (arrow) and (f) resolution of the sign (i.e., symmetrical signal of the pulvinar bilaterally or “equal”) post-resection.

Throughout the interval, 20 alterations to the hypointense pulvinar sign were noted. 10 patients changed from “incorrect” or “equivocal” to “correct” and included one AVM (embolized at interval), one cavernoma (resected at interval), one encephalomalacia (no interval change), one siderosis (no interval change), one giant aneurysm (no interval treatment), and five tumors (all resected at interval) (Figure 4). Conversely, in 10 cases, the findings went in the opposite direction; three from “correct” to “incorrect”: one encephalomalacia (no interval change), one cavernoma (no interval treatment), one MRI negative (no interval change) and seven to “equivocal”: five meningioma (all resected at interval) (Figure 5), one tumor (resected at interval), one cavernoma (resected at interval).

### Inter-rater reliability

Inter-rater reliability was substantial, Cohen’s kappa = 0.62 (*p* < 0.001).

## Discussion

In this study, we propose a novel visual MRI biomarker on SWI termed the “hypointense pulvinar sign”, and highlight its potential significance with regards to iron deposition in epilepsy. Among MRIs conducted at the time of epilepsy diagnosis, the hypointense pulvinar sign was correct in 44% of cases. Notably, right hemispheric lesions exhibited a significantly higher proportion of correct hypointense pulvinar signs compared to the left hemisphere (46% vs 24%). Siderosis and meningioma emerged as the most potent generators of a correctly lateralized hypointense pulvinar sign (71% and 54%, respectively). Interestingly, after resection, most cases with meningioma displaying initially correct hypointense pulvinar signs reverted to equal pulvinar hypointensity (5/7 cases).

The pulvinar’s metabolism of iron in epilepsy remains elusive and to the authors’ knowledge, no dedicated MRI studies on human subjects exist. The hippocampus has been extensively researched in this regard, where the seizure-induced cascade of events beginning with restricted diffusion and ending with iron deposition has been established.^17,34^ The hippocampus is more frequently implicated in seizure activity than is the pulvinar^35^ and despite the extensive pulvino-cortical connectivity,^11–14^ not all seizure activity results in pulvinar restricted diffusion. Factors such as prolonged seizure duration and occurrence of status epilepticus appear to influence the likelihood of pulvinar restricted diffusion.^2,36^

In a study on 10 patients with complex partial status epilepticus, Szabo et al. report nine cases of pulvinar restricted diffusion, all of which were found ipsilateral to the seizure focus based on MRI or EEG.^37^ Similarly, Rennebaum et al. reported ipsilateral pulvinar restricted diffusion in 12 of 69 patients with status epilepticus.^7^ In the largest study to date, Hübers et al. imaged 454 patients within 24 h of seizure onset ranging from single seizure to status epilepticus.^2^ Among these, only 18 patients exhibited restricted diffusion on MRI and only two displayed thalamic restricted diffusion (no further specification as to thalamic sublocation or correlation to status epilepticus). Regarding seizure origin, Capecchi et al. noted temporal status epilepticus as most frequently associated with pulvinar restricted diffusion, followed by parietal and only rarely in frontal status epilepticus.^14^ Furthermore, a significantly higher proportion of status epilepticus cases led to left-sided pulvinar restricted diffusion, likely due to stronger pulvino-cortical connectivity in the language dominant hemisphere.^38^

Following restricted diffusion, epileptiform activity in the hippocampus triggers abnormal uptake of iron by both neurons and astrocytes, kindling a pro-inflammatory state that contributes to increased neuronal loss and heightened long-term increased epileptogenicity.^17^ The epileptogenic effect of iron stems from increased lipid peroxidation, resulting in increased extracellular glutamate (an excitatory neurotransmitter) and reduced GABA_A_ (inhibitory neurotransmitter) receptor activity through protein function interference.^19,39,40^ While it is plausible that the same cascade of events may occur in the pulvinar, no previous studies have assessed pulvinar iron deposition prior to this work. Furthermore, the presence of restricted diffusion may not be necessary as pulvinar iron deposition could be more gradual, akin to the accelerated rate of cortical thinning observed in epilepsy patients. Current long-term pulvinar studies have focused on T2/FLAIR hyperintense signal change and atrophy, attributed to neuronal loss and reactive gliosis.^41–44^

To date, only studies focusing on dementia have explored hypointense signal change of the pulvinar on SWI, all hypothesizing increased pulvinar iron accumulation. In patients with Parkinson’s disease, pulvinar hypointensity on SWI correlated with cognitive decline after deep brain stimulation (DBS), a probable consequence of increased α-synuclein content.^31^ α-synuclein binds iron in the form of Fe(II) and Fe(III)^45–47^ and has been found elevated in the pulvinar of patients with dementia with Lewy bodies based on autopsy studies.^48^ Additionally, in Alzheimer’s patients, hypointense pulvinar signal change based on fluid attenuated inversion recovery (FLAIR) was correlated with alterations in T2* relaxometry, an MRI technique used for tissue iron quantification.^49^ Abnormal β-amyloid precipitation in Alzheimer’s disease has been associated with abnormally increased iron deposition.^40^

The MRI technique used in this study, SWI, is highly sensitive but is not specific for iron deposition and can be falsely positive due to the presence of calcium. Theoretically, SWI phase images can be used to discern between the two, but depending on the MRI vendor’s implementation of SWI, this information is not always available. While SWI from Siemens, for example, combines magnitude and phase information, the susceptibility-weighed SWAN sequence from GE relies solely on magnitude images and does not generate a phase map.^50^ These variations in sequence implementation lead to differing sensitivities and representations of iron relative to the underlying brain tissue. Furthermore, the complex ovoid shape of the pulvinar and intrinsically scattered foci of SWI hypointensity frequently result in inconclusive phase information, a phenomenon attributed to the high-pass filter and dipole effects.^50^

Our study has several limitations. First, our assessment of pulvinar hypointensity relied solely on visual assessment and quantitative analysis was not performed. The retrospective nature of our study precluded the use of QSM which is more accurate, but less widely available, than SWI for assessing brain iron deposition. Furthermore, substantial variations in sub-group sizes with respect to MRI scanner/sequence type and underlying brain lesion limited meaningful sub-analysis. Additionally, there were differences in the proportion of epilepsy patients scanned using SWI and SWAN sequences compared to healthy controls which may account for differences in hypointense pulvinar lateralization. This was due to different allocation of patients based on priority and differences in in-patient and out-patient MRI vendor availabilities at our institution. Finally, the reversion of the initially correctly lateralized sign after meningioma resection seen in 5/7 cases might suggest alternative mechanisms occurring within the pulvinar relating to SWI hypointensity. One such possibility is seizure-induced increased oxygen extraction leading to an increase in paramagnetic deoxyhemoglobin,^51^ which is reversible. The significantly higher number of right-sided hypointense pulvinar signs further corroborates the need to consider alternate mechanisms. This finding was unexpected considering Capecchi et al.’s results, where a significantly higher proportion of left-sided pulvinar restricted diffusion was found, albeit in patients with status epilepticus.^38^

In the end, the goal of our study was to evaluate the potential of the hypointense pulvinar sign as a visual biomarker in epilepsy patients. While the significance of this sign remains uncertain, we have shown it to be reproducible and simple to use, suggesting its suitability for inclusion into multimodal precision medicine models. Further quantitative research using QSM and assessment in multimodal predictive models correlated to patient outcome is required to better understand the sign’s significance. 

## Appendix

### Abbreviations

ADEM: Acute Demyelinating Encephalomyelitis
AVM: Arteriovenous Malformation
DBS: Deep Brain Stimulation
DWI: Diffusion Weighted Imaging
EEG: Electroencephalography
FLAIR: Fluid Attenuated Inversion Recovery
GABA_A_: γ-aminobutyric acid-A
MRI: Magnetic Resonance Imaging
MPRAGE: Magnetization Prepared Rapid Acquisition with Gradient Echoes
PACS: Picture Archiving and Communication System
QSM: Quantitative Susceptibility Mapping
SWAN: Susceptibility Weighted Angiography
SWI: Susceptibility Weighted Imaging
